# Characteristics and Outcomes of Older Patients Undergoing Protected Percutaneous Coronary Intervention With Impella

**DOI:** 10.1161/JAHA.124.038509

**Published:** 2025-04-16

**Authors:** Philipp Jakob, Alexandra J. Lansky, Mir B. Basir, Michael J. Schonning, Batla Falah, Zhipeng Zhou, Wayne B. Batchelor, Arsalan Abu‐Much, Cindy L. Grines, William W. O'Neill, Barbara E. Stähli

**Affiliations:** ^1^ Department of Cardiology, University Heart Center, University Hospital Zurich and the Center for Translational and Experimental Cardiology (CTEC) University of Zurich Zurich Switzerland; ^2^ Department of Cardiology Yale University School of Medicine New Haven CT USA; ^3^ Center for Structural Heart Disease, Division of Cardiology Henry Ford Health System Detroit MI USA; ^4^ Clinical Trials Center Cardiovascular Research Foundation New York NY USA; ^5^ Inova Center of Outcomes Research Inova Heart and Vascular Institute Falls Church VA USA; ^6^ Department of Cardiology Northside Hospital Cardiovascular Institute Atlanta GA USA

**Keywords:** age differences, all‐cause mortality, high‐risk percutaneous coronary intervention, major adverse cardiovascular and cerebrovascular events, PROTECT III, Percutaneous Coronary Intervention

## Abstract

**Background:**

In patients undergoing high‐risk percutaneous coronary intervention, Impella has become an important adjunctive tool to support revascularization. The impact of age on the outcomes of patients undergoing high‐risk percutaneous coronary intervention is limited. The aim of this study is to describe the characteristics and outcomes of patients ≥75 years of age undergoing Impella‐supported high‐risk percutaneous coronary intervention.

**Methods and Results:**

Baseline characteristics and outcomes of patients ≥75 years of age versus those of patients <75 years of age in patients enrolled in the cVAD PROTECT III (Catheter‐Based Ventricular Assist Device Prospective, Multi‐Center, Randomized Controlled Trial of the IMPELLA RECOVER LP 2.5 System Versus Intra Aortic Balloon Pump in Patients Undergoing Non Emergent High Risk Percutaneous Coronary Intervention) study (NCT04136392). Major adverse cardiovascular and cerebral events (composite of all‐cause death, nonfatal myocardial infarction, stroke/transient ischemic attack, and repeat revascularization) were assessed at 30 and 90 days and all‐cause death at 1 year. Out of 1237 patients, 493 (39.9%) patients were ≥75 years of age. Patients ≥75 years of age had less diabetes and prior myocardial infarction, more hypertension and dyslipidemia, worse renal function, more severe valvular heart disease, but higher left ventricular ejection fraction (*P*<0.05 for all comparisons). Baseline Synergy Between Percutaneous Coronary Intervention With Taxus and Cardiac Surgery scores were similar between groups. Older patients underwent more left main percutaneous coronary intervention (58% versus 39%; *P*<0.0001), atherectomy (32% versus 22%; *P*<0.0001), and femoral access (87% versus 79%, *P*=0.0003) as compared with younger patients. In‐hospital vascular complications did not differ, but rates of respiratory failure, pericardial tamponade, and cardiogenic shock were higher in older patients. Rates of all‐cause death and major adverse cardiovascular and cerebral events did not differ between groups at 30 and 90 days. Rates of all‐cause death at 1 year were higher in patients ≥75 years (adjusted hazard ratio, 1.99 [95% CI, 1.24–3.18], *P*=0.004).

**Conclusions:**

Impella‐supported high‐risk percutaneous coronary intervention in older patients is feasible with an acceptable safety profile. However, age ≥75 years remained a statistically significant predictor for all‐cause death at 1 year.

**Registration:**

URL: https://clinicaltrials.gov; Unique Identifier: NCT04136392.

**Nonstandard Abbreviations and Acronyms**
cVAD: catheter‐based ventricular assist device
HRPCI: high‐risk percutaneous coronary intervention
MACCE: major adverse cardiovascular and cerebrovascular events

**Clinical Perspective**

**What Is New?**

- Risk‐adjusted rates of death and major adverse cardiovascular and cerebral events at 30‐ and 90‐days post procedure were statistically not different in older patients (≥75 years) undergoing Impella‐supported high‐risk percutaneous coronary intervention as compared with younger patients.
- Notably, age ≥75 years was a statistically significant predictor of all‐cause death at 1 year.

**What Are the Clinical Implications?**

- Our results suggest that Impella support in older high‐risk patients undergoing percutaneous coronary intervention can be performed with comparable efficacy and an acceptable safety profile despite more extensive and complex coronary artery disease including left main/multivessel disease and calcified lesions.

An aging population coupled with an increasing prevalence of coronary artery disease (CAD)^1^, ^2^ has resulted in the increasing use of revascularization in older patients that can be safely performed.^3^, ^4^, ^5^, ^6^ Despite the higher risk profile of older patients, characterized by multiple comorbidities, frailty, and extensive CAD,^7^, ^8^ older patients undergoing high‐risk percutaneous coronary intervention (HRPCI) may derive equal or even greater benefit when compared with younger patients.^9^, ^10^, ^11^ However, high risk features combined with sparse clinical outcomes data may lead to underuse of both diagnostic testing for and treatment of CAD in older patients.^12^, ^13^ Greater disparities may occur when older patients present with impaired left ventricular (LV) systolic function and complex CAD.

Due to the extensive clinical and anatomical risk features, operators have to rely on a safety net to ensure hemodynamic stability. Current guidelines support the use of an Impella percutaneous LV assist device in selected patients undergoing HRPCI.^14^, ^15^ The randomized PROTECT II (Prospective, Randomized Clinical Trial of Hemodynamic Support With Impella 2.5 Versus Intra‐Aortic Balloon Pump in Patients Undergoing High‐Risk Percutaneous Coronary Intervention) trial,^16^ which investigated percutaneous LV assist devices in complex coronary scenarios and reduced LV ejection fraction (LVEF), had to be stopped early and narrowly failed to show a clinical benefit as compared with intra‐aortic balloon pump at the primary end point at 30 days in the intention‐to‐treat population. However, a prespecified analysis for the primary outcome at 90 days reached significant improvement in clinical outcomes in the per protocol analysis, leading to Food and Drug Administration approval for the Impella mechanical support device.

There is little evidence on the safety and efficacy of Impella‐supported HRPCI in older patients. This uncertainty may hinder interventional cardiologists from using percutaneous LV assist devices for HRPCI in such patients. The PROTECT III (Prospective, Multi‐Center, Randomized Controlled Trial of the IMPELLA RECOVER LP 2.5 System Versus Intra Aortic Balloon Pump in Patients Undergoing Non Emergent High Risk Percutaneous Coronary Intervention) trial is a Food and Drug Administration audited, prospective, multicenter, observational study that assessed outcomes of patients with complex CAD and severely depressed LV systolic function undergoing Impella‐supported HRPCI.^17^, ^18^ The PROTECT III trial demonstrated improved completeness of revascularization and outcomes with the use of contemporary Impella‐supported HRPCI when compared with the previous PROTECT II study.^16^, ^18^

The aim of this study was to assess outcomes of older patients undergoing Impella‐supported HRPCI in the contemporary PROTECT III study.

## METHODS

### Study Design and Patient Population

The PROTECT III study design has previously been published.^18^ Briefly, the PROTECT III trial is a multicenter, single‐arm, observational, Food and Drug Administration‐audited postapproval study nested within the Global cVAD (Catheter‐Based Ventricular Assist Device) Registry (NCT04136392),^17^ which aims to monitor postmarket safety and efficacy of the Impella 2.5/CP for HRPCI at 46 sites in the United States. The design of the Global cVAD study (NCT04136392) has been published previously.^17^ The cVAD registry supports multiple protocol‐driven postapproval studies designed in collaboration with the Food and Drug Administration for distinct indications and patient subgroups, one of which is the PROTECT III study. Inclusion and exclusion criteria have been previously reported.^19^

Baseline characteristics, preprocedural echocardiography, PCI, and data pertaining to the device were collected from time of admission through discharge. Angiographic data analysis (including the calculation of quantitative Synergy Between Percutaneous Coronary Intervention with Taxus and Cardiac Surgery (SYNTAX) and ischemia jeopardy scores) were conducted by the Beth Israel Deaconess Medical Center Angiographic Core Laboratory, and a Clinical Events Committee adjudicated all major adverse cardiovascular and cerebrovascular events (MACCE).

The registry was conducted in accordance with the Declaration of Helsinki, and institutional review board or independent ethics committees at each participating clinical site reviewed and approved the protocol before patient enrollment; thereby each patient gave informed consent. An independent 12‐member steering committee, including interventional cardiologists, cardiac surgeons, and heart failure specialists, oversaw the conduct of the cVAD registry. The sponsor (Abiomed, Danvers, MA) oversaw study data management and source document verification and provided funding to the Cardiovascular Research Foundation (New York, NY) for statistical analysis. Patients undergoing elective or urgent HRPCI procedures received either the Impella 2.5 or the Impella CP (Abiomed, Danvers, MA) and were treated in accordance with the hospital standard of care.^18^ The authors had unrestricted access to the study data and assume responsibility for the accuracy and reliability of this report. Artificial intelligence was not used in any step of the data curation, data analysis, or in writing of the article. The data that support the findings of this study are available upon reasonable request to the corresponding author.

### Study End Points and Definitions

This study assessed all‐cause death at 30 days, 90 days, and 1 year, and rates of MACCE, a composite of all‐cause death, nonfatal myocardial infarction (MI), stroke/transient ischemic attack (TIA), and repeat revascularization at 30 and 90 days. Detailed definitions of study end points have been previously published.^18^, ^19^

### Statistical Analysis

Baseline characteristics were presented using means±SD or median (interquartile range) for continuous variables, and percentages for categorical variables. Comparison between groups were conducted using *t* tests for continuous variables and chi‐square or Fisher's exact tests for categorical variables. For analyses regarding time to the first event, event rates were calculated using the Kaplan–Meier method and differences were examined using the log‐rank test. A multivariable Cox regression model for 1‐year all‐cause death was constructed to adjust for potential confounders. The variables included in the multivariable Cox regression (LVEF, estimated glomerular filtration rate, pre‐PCI SYNTAX score, and left main PCI) were selected based on prior evidence in the literature and their significance in the univariable analysis. All statistical analysis was 2 sided, and the analysis was performed using SAS software version 9.4 (SAS Institute Inc., Cary, NC).

## RESULTS

### Baseline Characteristics

Of 1237 patients enrolled in the PROTECT III study from March 2017 to March 2020, 493 (39.9%) were ≥75 years of age. Baseline characteristics are shown in Table 1.

**Table 1:** Baseline Characteristics

	Age <75 y (N=744)	Age ≥75 y (N=493)	*P* value
Demographics			
Age, y	63.8±7.7	81.9±4.7	<0.0001
Sex, female	24.9% (185/744)	29.8% (147/493)	0.054
Race			
White	62.4% (464/744)	74.4% (367/493)	<0.0001
Black	16.4% (122/744)	6.3% (31/493)	<0.0001
Asian	3.2% (24/744)	2.8% (14/493)	0.70
American Indian or Alaska Native	0.8% (6/744)	0% (0/493)	0.05
Native Hawaiian or Other Pacific Islander	0.1% (1/744)	0% (0/493)	0.42
Other	3.8% (28/744)	2.8% (14/493)	0.38
Unknown	13.3% (99/744)	13.6% (67/493)	0.89
Body mass index, kg/m^2^	29.6±7.0	27.2±5.3	<0.0001
Medical history			
Current/former smoker	65.4% (475/726)	57.2% (274/479)	0.004
Hypertension	90.2% (666/738)	94.3% (462/490)	0.01
Dyslipidemia	78.0% (573/735)	83.0% (404/487)	0.03
Diabetes	60.9% (451/740)	48.5% (237/489)	<0.0001
Stroke/transient ischemic attack	16.1% (118/735)	18.7% (91/487)	0.23
Prior myocardial infarction	42.8% (303/708)	36.9% (176/477)	0.04
Prior percutaneous coronary intervention	40.2% (293/729)	35.9% (175/488)	0.13
Prior coronary artery bypass graft	16.2% (119/735)	12.4% (61/493)	0.06
Chronic kidney disease	30.7% (226/735)	33.4% (163/488)	0.33
eGFR*, mL/min per 1.73 m^2^	74.0±24.9	59.6±21.5	<0.0001
Congestive heart failure	61.5% (452/735)	58.6% (285/486)	0.32
Prior pacemaker/cardiac resynchronization therapy/ implantable cardioverter‐defibrillator implantation	17.4% (119/685)	17.5% (80/457)	0.95
Anemia	19.5% (127/652)	20.2% (87/431)	0.77
Hemoglobin, g/dL	12.2±2.2	11.7±1.9	0.0002
Admission characteristics			
Angina	44.9% (300/668)	41.6% (182/437)	0.28
Acute myocardial infarction	35.5% (233/656)	34.8% (149/428)	0.81
STEMI	18.8% (44/234)	18.0% (27/150)	0.84
Non‐STEMI	80.8% (189/234)	81.3% (122/150)	0.89
Out‐of‐hospital cardiac arrest	1.6% (11/680)	2.2% (10/448)	0.45
Echocardiography characteristics			
Left ventricular ejection fraction (%)	32.0±14.5	37.7±15.9	<0.0001
Severe mitral valve regurgitation	4.2% (17/404)	5.1% (14/275)	0.59
Severe mitral valve stenosis	0.7% (2/287)	0% (0/200)	0.24
Severe aortic valve regurgitation	0.0% (0/373)	0.4% (1/259)	0.23
Severe aortic valve stenosis	3.4% (12/352)	13.5% (33/244)	<0.0001
Severe valvular disease†	7.2% (30/418)	16.6% (47/283)	<0.0001

Data are presented as mean±SD or % (n/N), where applicable. eGFR indicates estimated glomerular filtration rate; and STEMI, ST‐segment–elevation myocardial infarction.

* eGFR was calculated using 2021 Chronic Kidney Disease Epidemiology Collaboration Creatinine Equation.

^†^ Includes severe aortic stenosis/regurgitation and severe mitral stenosis/regurgitation.

Patients ≥75 years of age were more frequently White, tended to more often be female, had lower body mass index, and had more hypertension, dyslipidemia, less smoking, less diabetes, and fewer prior MIs. Estimated glomerular filtration rate and hemoglobin levels were lower in older patients. Older patients presented with similar rates of angina and acute MI compared with younger patients, with better LVEF by echocardiography, and a higher rate of severe valvular heart disease (Table 1).

### Angiographic and Procedural Characteristics

There was no difference in the number of diseased coronary arteries between groups and the majority of patients presented with 3‐vessel CAD (Table 2). At baseline, SYNTAX scores were similar between groups (28.5 versus 27.7, *P*=0.35) and left main disease was more prevalent in older patients. Older patients had more severe calcifications but more focal lesions (Table S1).

**Table 2:** Procedural Characteristics

	Age <75 y (N=744)	Age ≥75 y (N=493)	*P* value
Coronary angiography			
Left main disease	52.1% (384/737)	68.4% (335/490)	<0.0001
Number of diseased vessels			0.82
1	10.8% (80/744)	11.8% (58/493)	0.58
2	30.4% (226/744)	30.4% (150/493)	0.99
3	56.6% (421/744)	55.0% (271/493)	0.58
>3	1.5% (11/738)	1.8% (9/488)	0.63
SYNTAX score	27.7±12.4	28.5±12.6	0.35
Ischemia jeopardy score	8.7±2.2	9.1±2.0	0.02
PCI			
Number of vessels treated			0.62
1	30.5% (227/744)	28.4% (140/493)	0.43
2	45.0% (335/744)	44.6% (220/493)	0.89
3	24.2% (180/744)	26.6% (131/493)	0.35
Left main lesion treated	38.8% (289/744)	58.4% (286/490)	<0.0001
Number of lesions treated	2.6±1.5	2.4±1.3	0.009
Adjunct procedural diagnostics	65.4% (462/706)	72.4% (343/474)	0.01
Atherectomy	54.5% (252/462)	70.0% (240/343)	<0.0001
Optical coherence tomography/intravascular ultrasound	73.7% (322/437)	68.4% (219/320)	0.11
Fractional flow reserve	3.7% (16/437)	1.9% (6/320)	0.15
Temporary pacer	10.3% (45/437)	18.4% (59/320)	0.001
PCI arterial access			
Radial	19.5% (134/688)	12.6% (57/453)	0.002
Femoral	78.6% (541/688)	87.0% (394/453)	0.0003
Contrast volume, mL	210.4±107.5	196.9±102.4	0.03
Post‐PCI SYNTAX score	6.9±8.2	5.8±8.0	0.04
Post‐PCI ischemia jeopardy score	2.1±2.2	1.8±2.1	0.04
Impella support			
Device			
Impella 2.5	31.5% (234/744)	28.2% (139/493)	0.22
Impella CP	68.5% (510/744)	71.8% (354/493)	0.22
Successful implantation	99.6% (741/744)	99.6% (491/493)	0.99
Impella access			
Femoral artery	94.1% (700/744)	95.1% (469/493)	0.43
Subclavian/transaxillary/transcaval	5.9% (44/744)	4.3% (21/493)	0.20

Data are presented as mean±SD or % (n/N), where applicable. PCI indicates percutaneous coronary intervention; and SYNTAX, Synergy Between Percutaneous Coronary Intervention With Taxus and Cardiac Surgery.

In older patients, radial access was less common, and fewer lesions were treated, but they had more left main PCI (58.4% versus 38.8%; *P*<0.0001) and were more often treated with atherectomy (32.4% versus 21.6% [on lesion level]; *P*<0.0001, Table 2 and Table S1). The post‐PCI residual SYNTAX score and post‐PCI ischemia jeopardy score were lower in older patients (Table 2). In‐hospital events, including cardiac perforation, cardiogenic shock, respiratory and renal dysfunction, and bleeding (Bleeding Academic Research Consortium score ≥3a) were higher in older patients (Table S2). Assessment of patients across age quartiles revealed that complications are more likely to occur in patients >80 years (Table S3).

### Outcomes at 30 and 90 Days

**Figure 1:**
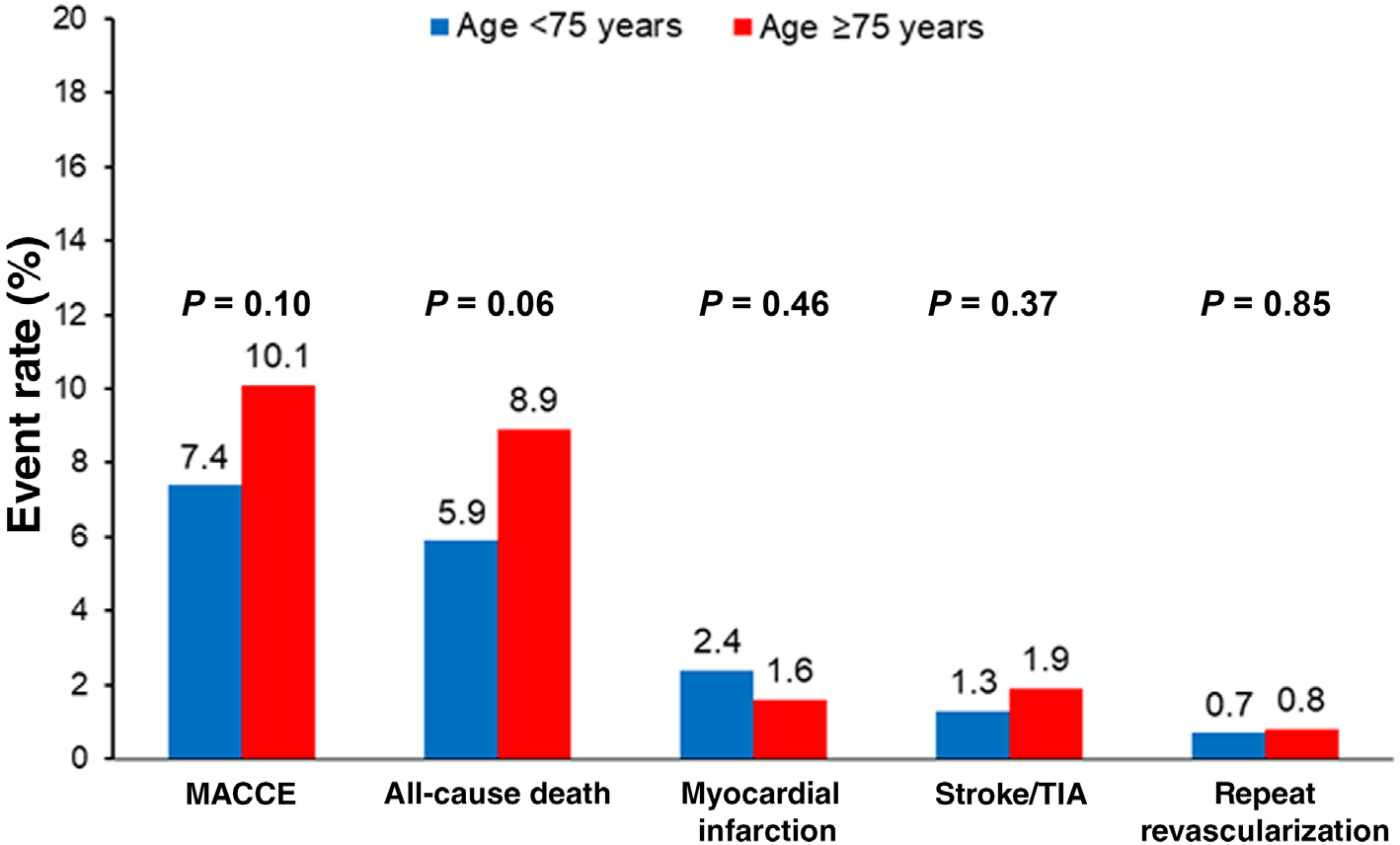
Kaplan–Meier event rates at 30 da stratified by age (</≥75 years). MACCE is the composite of all‐cause death, myocardial infarction, stroke/TIA, and repeat revascularization. MACCE indicates major adverse cardiac and cerebrovascular events; and TIA, transient ischemic attack.

There were no significant differences in rates of MACCE at 30 days between the groups (10.1% versus 7.4%; HR: 0.71; CI: 0.47–1.07; *P*=0.10, Figure 1 and Table S4). Rates of all‐cause death, although numerically higher in older patients, did not differ between groups (8.9% versus 5.9%; hazard ratio [HR], 0.65 [95% CI, 0.41–1.03]; *P*=0.06). Rates of cardiovascular (7.7% versus 5.5%, *P*=0.17) and noncardiovascular death (1.3% versus 0.4%, *P*=0.08) were similar between groups (Table S3). Rates of nonfatal MI, stroke/TIA, and repeat revascularization were low and did not differ between groups (Figure 1 and Table S4).

**Figure 2:**
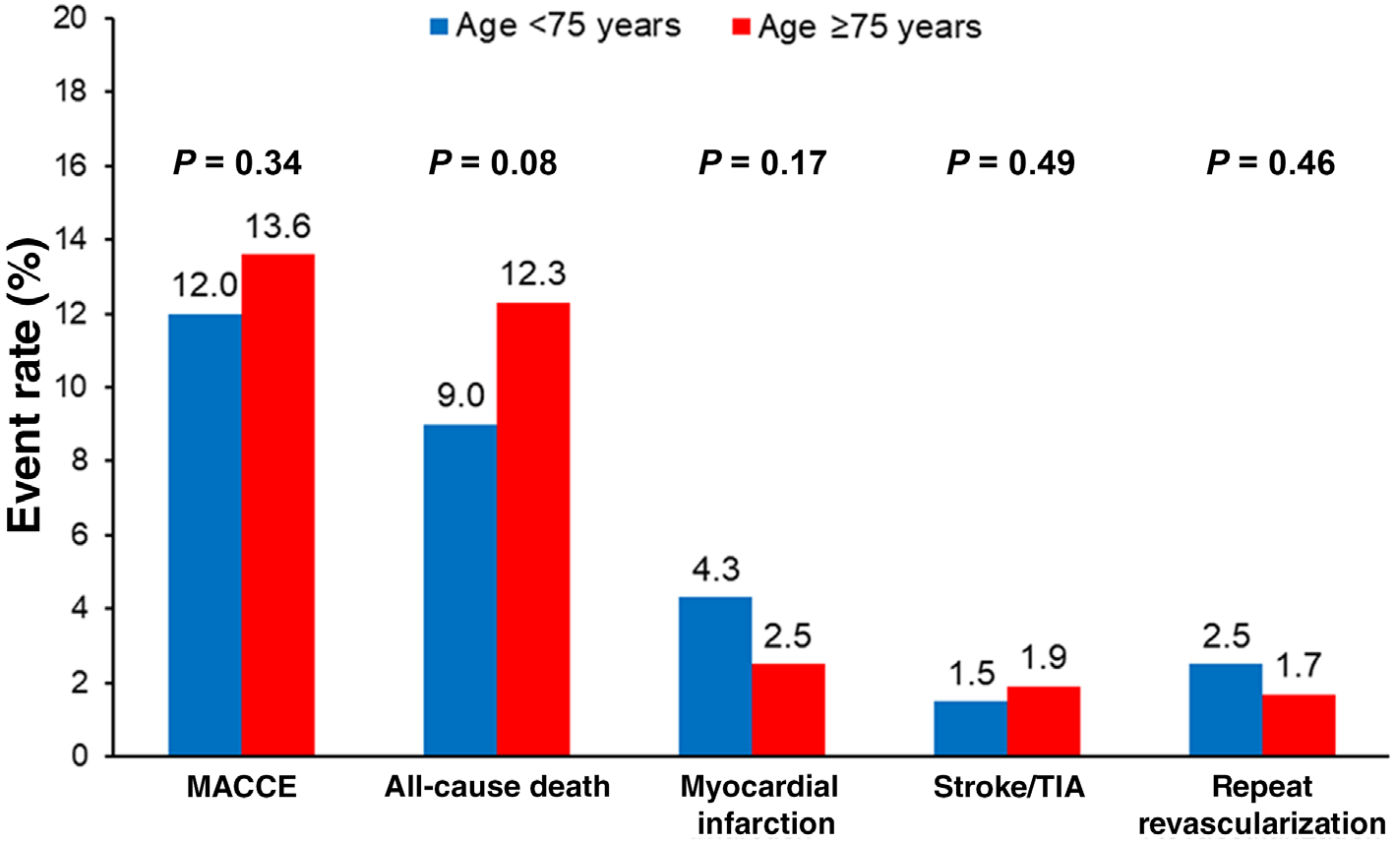
Kaplan–Meier event rates at 90 days stratified by age (</≥75 years). MACCE is the composite of all‐cause death, myocardial infarction, stroke/TIA, and repeat revascularization. MACCE indicates major adverse cardiac and cerebrovascular events; and TIA, transient ischemic attack.

At 90 days, there was no difference in rates of MACCE (13.6% versus 12.0%; HR, 0.84 [95% CI, 0.60–1.19]; *P*=0.34) between groups (Figure 2). Similarly, all‐cause death (12.3% versus 9.0%; HR, 0.71 [95% CI, 0.48–1.04]; *P*=0.08), nonfatal MI, stroke/TIA, and repeat revascularization did not differ between groups (Figure 2 and Table S5).

### All‐Cause Death at 1 Year

**Figure 3:**
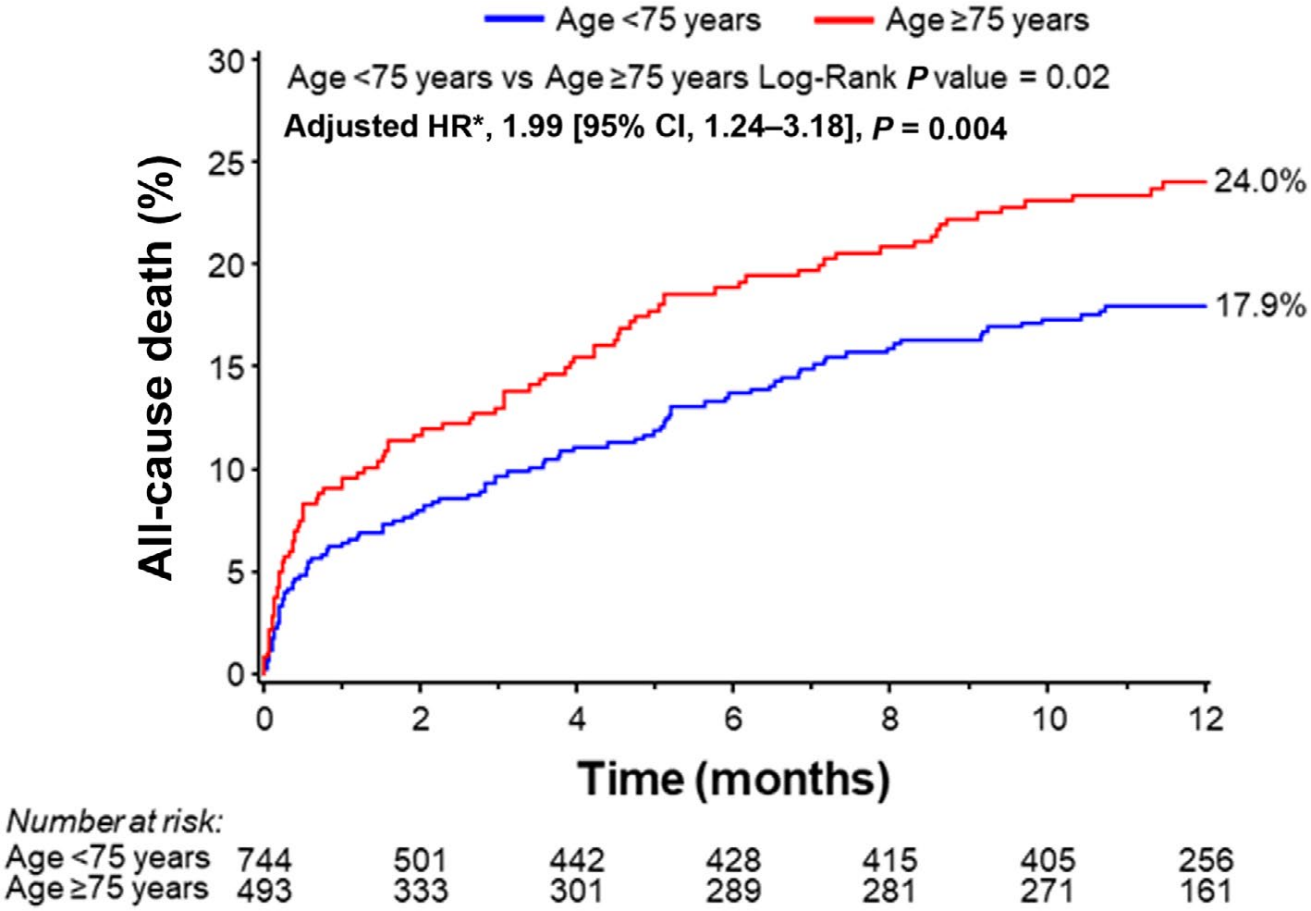
Kaplan–Meier event rates through 1 year stratified by age (</≥75 years). *Multivariable adjustment for estimated glomerular filtration rate, left ventricular ejection fraction, left main percutaneous coronary intervention, and pre‐percutaneous coronary intervention SYNTAX score. HR indicates hazard ratio.

At 1 year, older patients had a higher rate of all‐cause death as compared with younger patients (24.0% versus 17.9%; HR, 1.40 [95% CI, 1.05–1.86]; *P*=0.02). This association remained significant after multivariable adjustment for estimated glomerular filtration rate, LVEF, left main PCI, and pre‐PCI SYNTAX score (HR, 1.99 [95% CI, 1.24–3.18]; *P*=0.004, Figure 3). Univariable and multivariable HRs by Cox regression for 1‐year all‐cause mortality are shown in Table S6. Categorization of patients into quartiles showed that patients >80 years had the highest rate of all‐cause death (Table S7).

## DISCUSSION

In this study, we investigated outcomes of Impella‐supported HRPCI in a large population of older patients with complex CAD and LV dysfunction, using data from the prospective, multicenter PROTECT III registry. The present study demonstrates for the first time that, in patients ≥75 years, Impella‐supported HRPCI can be performed with favorable outcomes. Rates of all‐cause death and MACCE, both at 30 and 90 days following Impella‐supported HRPCI, were not statistically different between age groups. Although clinical outcomes at 90 days illustrated the feasibility and efficacy with an acceptable safety profile of Impella‐supported HRPCI in older patients, the risk of all‐cause death at 1 year was higher as compared with younger patients. Hence, this study supports a strategy of protected HRPCI in older patients but also emphasizes the increased long‐term mortality in older patients.

### Revascularization Strategies, Impella Support, and Outcomes in Older Patients

As life expectancy continues to rise, cardiologists will face the growing challenge of treating older patients with more extensive CAD. Both, surgical and percutaneous revascularization may be reasonable options in this patient population. In the SYNTAX Extended Survival study, 5‐year MACCE and 10‐year mortality did not significantly differ between PCI and coronary artery bypass graft in the older population (age >70 years), suggesting that PCI is a valuable option in older patients with complex CAD.^20^ The SYNTAX II study, incorporating contemporary PCI techniques with functional lesion assessment and intravascular imaging, further showed improved outcomes of patients with 3‐vessel CAD in comparison to patients treated with PCI in the original SYNTAX I trial^21^, ^22^ and outcomes after PCI in older patients seem to improve over time.^23^ However, older patients undergoing PCI are at an increased risk of major events in the short and long term. Batchelor et al. showed that octogenarians have a more than 3‐fold higher rate of in‐hospital mortality as compared with younger patients,^23^ and differences were largely driven by cardiac and extracardiac comorbidities, such as LV systolic dysfunction and renal failure.^23^ In the SYNTAX Extended Survival study, PCI in patients >70 years had a more than 2‐fold higher rate of all‐cause mortality at 10‐year follow‐up as compared with younger patients.^20^ However, the low prevalence of patients with concomitant LV dysfunction in these studies precludes extrapolation to patients with advanced CAD and LV dysfunction, and older patients are constantly underrepresented in trials investigating Impella‐supported HRPCI.^16^

This study showed that short‐term rates of mortality and MACCE in patients ≥75 years undergoing Impella‐supported HRPCI were not statistically different as compared with younger patients, suggesting a similar efficacy profile of Impella support for revascularization in older patients at 30 and 90 days. There was a trend toward increased rates of death that are explained by a higher prevalence of both cardiac factors such as severe valvular heart disease and noncardiac comorbidities in older patients. These findings are in line with a previous substudy of the PROTECT II trial, which found no differences in rates of major adverse events, MACCE, in octogenarians as compared with younger patients.^24^ Here, we extend these findings to a large population of older patients undergoing Impella‐supported HRPCI, using data from the large, contemporary PROTECT III study.^18^ Although baseline SYNTAX scores did not differ between older and younger patients in this population, patients ≥75 years of age more frequently presented with left main disease and severely calcified lesions, suggesting a higher lesion complexity. This is also reflected by an increased use of atherectomy in older patients. The radial access was less often used in older patients. Despite these angiographic and procedural high‐risk features in older patients, rates of all‐cause death and MACCE did not significantly differ at 30 and 90 days. These favorable early clinical outcomes may be in part attributable to a greater reduction in SYNTAX score and support the feasibility of Impella‐supported HRPCI in older patients. Moreover, the low residual SYNTAX score reflects advanced contemporary interventional practice and illustrates complete revascularization strategies. This finding is important as several studies have suggested improved clinical outcomes in patients with complete revascularization,^25^, ^26^ accompanied by an increase in LV systolic function,^27^ that may be particularly favorable in the older population undergoing HRPCI. However, whereas PCI‐related complications were comparable between groups, ventricular perforation, cardiogenic shock, respiratory and renal dysfunction, and bleeding (Bleeding Academic Research Consortium score ≥3a) were higher in patients ≥75 years of age. Further analysis according to age quartiles indicate that complications are more likely to occur in patients >80 years. Despite advancement in interventional techniques, including larger experience with HRPCI and vascular access, the higher complication rate has to be taken into consideration before opting for Impella‐supported HRPCI. Importantly, vascular complications and limb ischemia that may be related to placement of the Impella device were similar in older patients. However, mechanical circulatory support did not blunt severe complications such as respiratory failure and cardiogenic shock that are potentially associated with a higher lesion complexity, severe valvulopathy, and noncardiac comorbidities in older patients. Integration of potential complications associated with comorbidities are therefore important for short‐ and long‐term risk assessment and older patients have to be informed of this additional risk burden when undergoing Impella‐supported HRPCI.

Patients with ischemia‐driven LV systolic dysfunction are at increased risk for mortality. Rates of all‐cause death at 1 year were higher in our analysis as observed in randomized trials and meta‐analysis in patients with reduced LVEF.^28^, ^29^, ^30^ However, patients in the PROTECT III study were older, with one third presenting with MI, and had a high proportion of left main stem and calcified lesions with use of ablative therapies. This unique combination of high‐risk features explains the high overall death burden. Compared with younger patients, rates of all‐cause death were higher after 1 year in patients ≥75 years and age was a statistically significant predictor of all‐cause death in multivariable analysis. The increased rates of all‐cause death in the older patients is in line with previous studies that investigated PCI in this patient population.^31^, ^32^, ^33^, ^34^ Besides the extent of CAD and the reduced LV systolic function that both contribute to a high mortality rate,^35^, ^36^ older patients have more comorbidities^37^ that are potentially associated with a shift from cardiovascular to noncardiovascular death. Indeed, in our study, older patients had worse renal function and a higher prevalence of severe valvular heart disease. Additional prognostic factors determining long‐term outcomes but not incorporated into the multivariable models may come into play in an older patient population. As recently shown, a higher mortality rate in older patients was also observed in patients with cardiogenic shock.^38^ Frailty that was not assessed in this registry, and surgical turndown, especially in patients >80 years with the highest observed mortality, may also have contributed to a higher rate of all‐cause death at 1 year.^7^, ^39^ Moreover, prescription rates and adherence to medical therapy are overall lower in older patients,^20^ which may be of particular importance in patients with heart failure.

### Limitations

Some limitations need to be considered. First, no uniform definition for an age threshold to stratify older patients from younger patients exists. Therefore, results have to be interpreted with caution when comparing with other studies using different age thresholds. Second, there is a lack of a universally accepted definition of HRPCI. In PROTECT III, the decision to use Impella‐supported HRPCI was left to the discretion of the operator. An expert consensus statement has proposed 3 domains for patients with CAD at high risk, comprising complex coronary anatomy, comorbidities, and adverse hemodynamics. In PROTECT III, a vast majority of patients had 1 or more risk factors in each domain (83%).^40^ However, further characterization of this high‐risk patient population is necessary that will be derived from large future randomized trials (NCT04763200, NCT05003817).^41^ Third, this study assessed characteristics and outcomes of older patients undergoing Impella‐supported HRPCI using prospective registry data, with the limitations inherent to such a design. However, registry data often reflect real‐world clinical practice and mostly include a broader patient population at increased risk as compared with randomized trials. As a next step, randomized trials are needed to define optimal treatment strategies for older patients with complex CAD and to compare outcomes of optimal medical management alone versus PCI with and without percutaneous LV assist device support. Third, residual confounding by factors not included in the multivariable models cannot be excluded completely and may have affected the results.

## CONCLUSIONS

In this study enrolling a large cohort of older patients, Impella‐supported HRPCI was feasible with an acceptable safety profile and short‐term outcomes did statistically not differ when compared with younger patients. Age, however, was a statistically significant predictor of all‐cause death at 1 year in this high‐risk population with complex CAD and LV dysfunction.

## Sources of Funding

The PROTECT III study, as part of the Global cVAD study, was sponsored by Abiomed Inc. (Danvers, MA, USA).

## Disclosures

Dr Lansky has received speaker fees from Abiomed. Dr Basir reports consultant/speaker fees from Abiomed, Boston Scientific, Chiesi, Saranas, and Zoll. Dr Batchelor has received speaker honoraria from Boston Scientific, Abbott Medical, and Medtronic. Dr Grines reports participation on the advisory boards for Philips and Abiomed. Dr O'Neill reports grant/research support from St Jude Medical, Edwards Life Sciences, and Biomed; consulting fees/honoraria from Medtronic and Abiomed; and major stock shareholder/equity in Synecor, Accumed, Neovasc, Tendyne, and Mitralign. Dr Stähli and her research has been supported by a donation of H.H. Sheikh Khalifa bin Hamad Al‐Thani to the University of Zurich, Switzerland; investigator sponsored research received at the University of Zurich from Boston Scientific and Edwards Lifesciences; research grant support received at the University of Zurich from the OPO Foundation, the Iten‐Kohaut Foundation, the German Center for Cardiovascular Research, the German Heart Research Foundation, and the B. Braun Foundation; speaker fees from Boston Scientific, Abbott Vascular, and MedAlliance; and is a Steering Committee Member of the Cruz Senior study (SMT). All other coauthors have no relevant disclosures.

## Supporting information

Tables S1–S7
