# Supplementary material for: Characteristics and Outcomes of Older Patients Undergoing Protected Percutaneous Coronary Intervention With Impella
Source: J Am Heart Assoc. 2025 Apr 16;14(9):e038509. doi: 10.1161/JAHA.124.038509 (PMC12184249; doi:10.1161/JAHA.124.038509)
Supplement: Supplementary file 1 — Tables S1–S7 [file JAH3-14-e038509-s001.pdf]

SUPPLEMENTAL MATERIAL

Table S1. Angiographic and Procedural Data (Lesion-Level Analysis).

	Age <75 years	Age ≥75 years	*P* Value
Degree of Calcification			
None/Mild	40.6% (653/1607)	23.3% (246/1055)	<0.0001
Moderate	17.2% (276/1607)	18.9% (199/1055)	0.28
Severe	42.2% (678/1607)	57.8% (610/1055)	<0.0001
Lesion Length, mm	15.3 ± 12.9	12.6 ± 11.1	<0.0001
Vessel Location (Treated Lesion)			
LM	13.7% (313/2288)	21.7% (316/1457)	<0.0001
LAD	39.0% (893/2288)	36.8% (536/1457)	0.18
LCX	28.2% (645/2288)	25.5% (371/1457)	0.07
RCA	16.6% (379/2288)	13.2% (192/1457)	0.005
Graft	2.5% (58/2288)	2.9% (42/1457)	0.53
Left Internal Mammary Artery	33.9% (19/56)	23.8% (10/42)	0.37
Saphenous Vein Graft	66.1% (37/56)	76.2% (32/42)	0.37
Lesion Location			
Proximal	42.4% (781/1841)	43.0% (526/1223)	0.77
Middle	32.2% (592/1841)	28.2% (345/1223)	0.02
Distal	18.8% (347/1841)	17.8% (218/1223)	0.51
Ostial	6.6% (121/1841)	11.0% (134/1223)	<0.0001
Atherectomy*	21.6% (498/2304)	32.4% (474/1464)	<0.0001

Data are presented as mean ± standard deviation, or % (n/N), where applicable. *rotational, orbital, and laser atherectomy. LAD denotes left anterior descending artery; LCX, left circumflex artery; LIMA denotes left internal mammary artery; LM, left main; RCA, right coronary artery; SVG, saphenous vein graft.

Table S2. In-hospital events.

	Age <75	Age ≥75	*P* Value
Cardiac perforation	0% (0/743)	1.4% (7/493)	0.001
Pericardial effusion with tamponade and drainage	0.4% (3/743)	2.2% (11/493)	0.003
Cardiogenic shock	1.6% (12/743)	4.1% (20/493)	0.008
Acute renal dysfunction	3.1% (23/743)	6.3% (31/493)	0.007
Life-threatening, disabling, or major bleeding (BARC≥3a)	1.7% (13/743)	3.7% (18/493)	0.04
Hemolysis	1.2% (9/743)	1.0% (5/493)	0.75
Anemia requiring transfusion	6.7% (50/743)	9.3% (46/493)	0.09
Major vascular or cardiac structural complications	0.5% (4/743)	0.4% (2/493)	0.74
Vascular complication requiring planned surgery	1.3% (10/743)	0.6% (3/493)	0.21
Vascular/cardiac structural complication requiring surgery/re-intervention	1.9% (14/743)	1.0% (5/493)	0.22
Vascular complication without surgery	1.5% (11/743)	1.4% (7/493)	0.93
Hematoma	6.9% (51/743)	8.1% (40/493)	0.41
Limb ischemia	1.9% (14/743)	1.6% (8/493)	0.73
Pulmonary embolism	0% (0/743)	0.2% (1/493)	0.22
Respiratory dysfunction/failure	0.8% (6/743)	3.0% (15/493)	0.003

Data are presented as mean ± standard deviation, or % (n/N), where applicable. BARC denotes Bleeding Academic Research Consortium.

Table S3. In-hospital events according to quartiles.

	Age <63	63≤ Age <72	72≤ Age <80	Age ≥80	*P* Value
Cardiac perforation	0% (0)	0% (0)	0.6% (2)	1.6% (5)	0.02
Pericardial effusion with tamponade and drainage	0% (0)	0.6% (2)	1.3% (4)	2.5% (8)	0.02
Cardiogenic shock	1.4% (4)	1.2% (4)	2.9% (9)	3.2% (10)	0.23
Acute renal dysfunction	3.9% (11)	2.8% (9)	4.8% (15)	5.7% (18)	0.29
Life-threatening, disabling, or major bleeding (BARC≥3a)	1.4% (4)	1.5% (5)	3.8% (12)	3.2% (10)	0.14
Anemia requiring transfusion	5.6% (16)	6.5% (21)	8.9% (28)	9.9% (31)	0.16
Vascular/cardiac structural complication requiring surgery/re-intervention	0% (0)	1.2% (4)	0.3% (1)	0.3% (1)	0.14
Vascular complication requiring planned surgery	1.1% (3)	1.5% (5)	1.3% (4)	0.3% (1)	0.47
Vascular complication without surgery	1.4% (4)	0.3% (1)	2.6% (8)	1.5% (5)	0.13
Limb ischemia	1.8% (5)	2.2% (7)	2.2% (7)	1.0% (3)	0.60
Respiratory dysfunction/failure	0.7% (2)	1.2% (4)	1.0% (3)	3.8% (12)	0.009

Data are presented as Kaplan Meier event rates (number of events). BARC denotes Bleeding Academic Research Consortium.

Table S4. Clinical Outcomes at 30 days.

	Age <75 years	Age ≥75 years	HR (95% CI)	*P* Value
MACCE	7.4% (48)	10.1% (44)	0.71 (0.47, 1.07)	0.10
All-cause Death	5.9% (37)	8.9% (37)	0.65 (0.41, 1.03)	0.06
Non-Cardiovascular Death	0.4% (2)	1.3% (5)	0.26 (0.05, 1.34)	0.08
Cardiovascular Death	5.5% (35)	7.7% (32)	0.71 (0.44, 1.15)	0.17
Myocardial Infarction	2.4% (15)	1.6% (7)	1.40 (0.57, 3.44)	0.46
Stroke/TIA	1.3% (9)	1.9% (9)	0.65 (0.26, 1.65)	0.37
Repeat Revascularization	0.7% (4)	0.8% (3)	0.87 (0.19, 3.87)	0.85

Data are presented as Kaplan Meier event rates (number of events). MACCE denotes major adverse cardiac and cerebrovascular event (all-cause death, myocardial infarction, stroke/TIA, and revascularization); TIA, transient ischemic attack.

Table S5. Clinical Outcomes at 90 days.

	Age <75 years	Age ≥75 years	HR (95% CI)	*P* Value
MACCE	12.0% (73)	13.6% (57)	0.84 (0.60, 1.19)	0.34
All-cause Death	9.0% (54)	12.3% (50)	0.71 (0.48, 1.04)	0.08
Non-Cardiovascular Death	0.6% (3)	1.9% (7)	0.28 (0.07, 1.08)	0.05
Cardiovascular Death	8.5% (51)	10.7% (43)	0.78 (0.52, 1.17)	0.22
Myocardial Infarction	4.3% (25)	2.5% (10)	1.65 (0.79, 3.44)	0.17
Stroke/TIA	1.5% (10)	1.9% (9)	0.73 (0.30, 1.79)	0.49
Repeat Revascularization	2.5% (13)	1.7% (6)	1.44 (0.55, 3.78)	0.46

Data are presented as Kaplan Meier event rates (number of events). MACCE denotes major adverse cardiac and cerebrovascular event (all-cause death, myocardial infarction, stroke/TIA, and revascularization); TIA, transient ischemic attack.

Table S6. Univariable and multivariable hazard ratios by Cox regression analysis for all-cause death through one year.

	Univariable Analysis		Multivariable Analysis	
Variable	HR (95% CI)	P Value	Adjusted HR (95% CI)	P Value
Age ≥75 years	1.40 (1.05, 1.86)	0.02	1.99 (1.24, 3.18)	0.004
Sex, male	0.76 (0.56, 1.03)	0.07		
Diabetes mellitus	1.27 (0.95, 1.70)	0.11		
eGFR*, mL/min/1.73 m^2	0.99 (0.98, 0.99)	0.0001	0.99 (0.98, 1.00)	0.07
LVEF, %	0.99 (0.98, 1.00)	0.034		0.0007
Severe Valvular Disease†	1.55 (0.96, 2.51)	0.07		
SYNTAX Score	1.02 (1.01, 1.03)	0.005	1.02 (1.00, 1.03)	0.049
LM-PCI	1.50 (1.13, 2.00)	0.006	1.44 (0.90, 2.31)	0.13
Prior myocardial infarction	1.01 (0.76, 1.36)	0.93		
Body mass index, kg/m^2	0.98 (0.96, 1.01)	0.14		

*eGFR was calculated using 2021 CKD-EPI Creatinine Equation. †Includes severe mitral regurgitation, mitral stenosis, aortic regurgitation, and aortic stenosis. eGFR denotes estimated glomerular filtration rate; LM-PCI, left main percutaneous coronary intervention; LVEF, left ventricular ejection rate; SYNTAX, Synergy Between PCI With Taxus and Cardiac Surgery.

Table S7. Kaplan Meier event rates through one year according to age quartiles.

	Age <63	63≤ Age <72	72≤ Age <80	Age ≥80	Combined	*P* Value
All-cause death	16.2% (33)	18.0% (44)	21.4% (51)	25.5% (63)	20.4% (191)	0.04
					**Relative Risk**	***P* Value**
**Age <63 vs. 63 ≤Age < 72**					0.86 (0.55, 1.34)	0.50
**Age <63 vs. 72 ≤Age <80**					0.70 (0.45, 1.09)	0.12
**Age <63 vs. Age ≥80**					0.58 (0.38, 0.88)	0.010
**63 ≤Age <72 vs. 72 ≤ Age < 80**					0.82 (0.55, 1.23)	0.35
**63 ≤Age <72 vs. Age ≥80**					0.67 (0.46, 0.99)	0.04
**72 ≤Age <80 vs. Age ≥80**					0.82 (0.57, 1.18)	0.28

Data are presented as Kaplan Meier event rates (number of events). Age reported as number of years.
